# A Rare Case of Angina Pectoris with the Longest Ectopic Left Main Coronary Artery Arising from Right Sinus of Valsalva and a Prepulmonic Course

**DOI:** 10.1155/2017/5483257

**Published:** 2017-01-15

**Authors:** Santosh Kumar Sinha, Vikas Mishra, Nasar Abdali, Karandeep Singh, Mukesh Jitendra Jha, Ashutosh Kumar

**Affiliations:** Department of Cardiology, LPS Institute of Cardiology, G.S.V.M. Medical College, Kanpur, Uttar Pradesh 208002, India

## Abstract

Knowledge of the morphoanatomical characteristics of the main trunk of the left coronary artery as well as its variations is cornerstone of hemodynamic, correct interpretation of coronary angiogram and for revascularization purpose. The left main coronary artery (LMCA) ranges from 3 to 6 mm in diameter and may be up to 10 to 15 mm in length in humans. We here report a case of the longest* anomalous* LMCA (56 mm) reported so far in a 35-year-old man with chronic stable angina arising from right sinus of valsalva as seen on conventional angiogram and multidetector computerized tomogram (MDCT).

## 1. Introduction

The subject of coronary artery anomalies (CAAs) has undergone profound evolutionary changes related to the definition, morphogenesis, clinical presentation, diagnostic workup, prognosis, and treatment of these anomalies [1, 2]. About the size of the cigarette butt, the left main coronary artery is a relatively small vessel, yet it is unarguably the most valuable section among the coronary angiogram. It emerges from the aorta through the ostia of the left aortic cusp, within the left sinus of valsalva. It travels from the aorta and passes between the pulmonary trunk and the left atrial appendage, under which it divides into left anterior descending artery and the left circumflex artery. Various anomalies have been described regarding its length, site of origin, and its course.

## 2. Case Report

**Figure 1:**
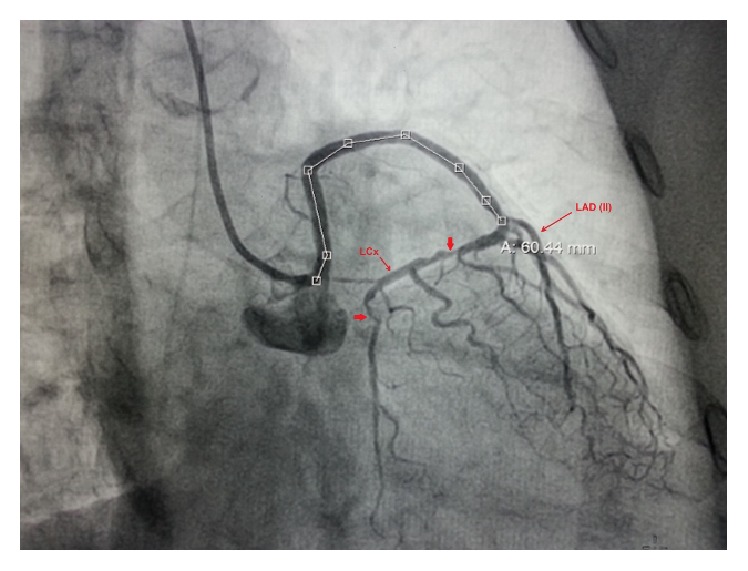
LMCA arising from right sinus revealing type II LAD and critical stenosis in LCx in AP cranial view.

A 35-year-old man, diabetic and hypertensive, presented with exertional angina CCS III despite guideline directed medical therapy. He was 153 cm tall and weighed 52 kg. His vitals were stable and cardiovascular system examinations were within normal limit. An electrocardiogram showed mild ST-T changes. His treadmill test was strongly positive for stress induced myocardial ischemia. Echocardiography revealed normal LV systolic function with an ejection fraction of 60% and trivial mitral regurgitation. Coronary angiogram was performed after proper consent through right radial route. We probed for left main coronary artery from its usual site but it was not visualized even on nonselective injection indicating anomalous origin. Finally it was selectively cannulated from right sinus giving type II LAD (left anterior descending artery) and LCx (left circumflex artery). There was discrete eccentric lesion with critical stenosis (type A lesion) in proximal and distal LCx responsible for his symptoms (Figure 1).

**Figure 2:**
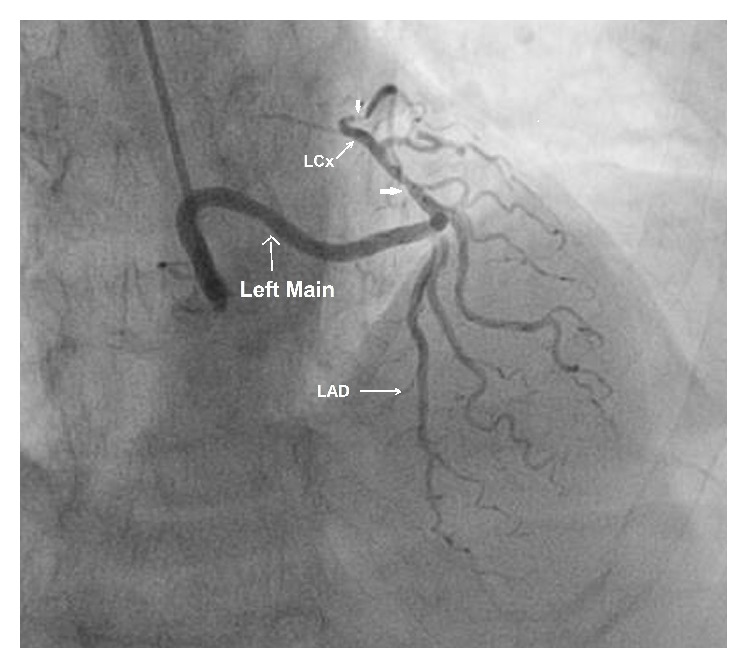
LMCA arising from right sinus revealing type II LAD and critical stenosis in LCx in AP caudal view.

**Figure 3:**
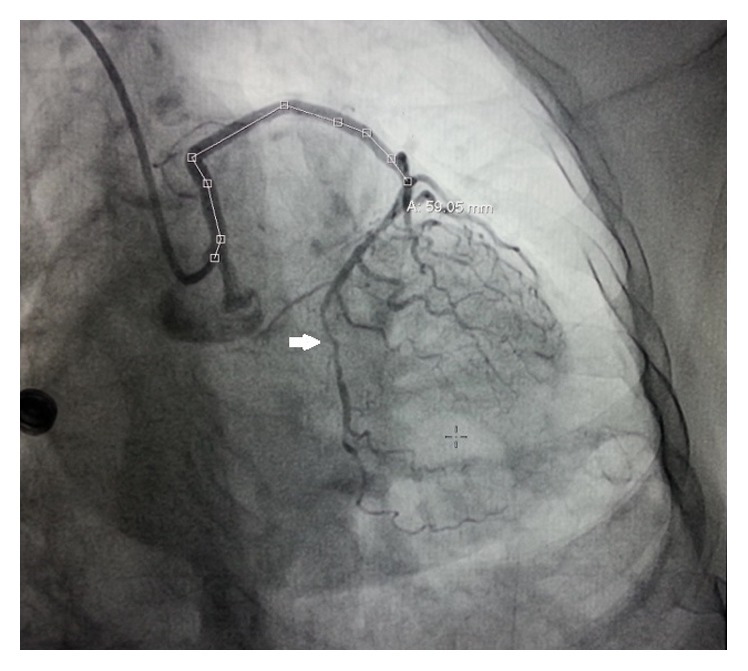
LMCA arising from right sinus revealing type II LAD and critical stenosis in LCx in AP caudal view.

It measured 59-60 mm in length in various projections (Figures 2 and 3) by quantitative coronary analysis software (QCA).

**Figure 4:**
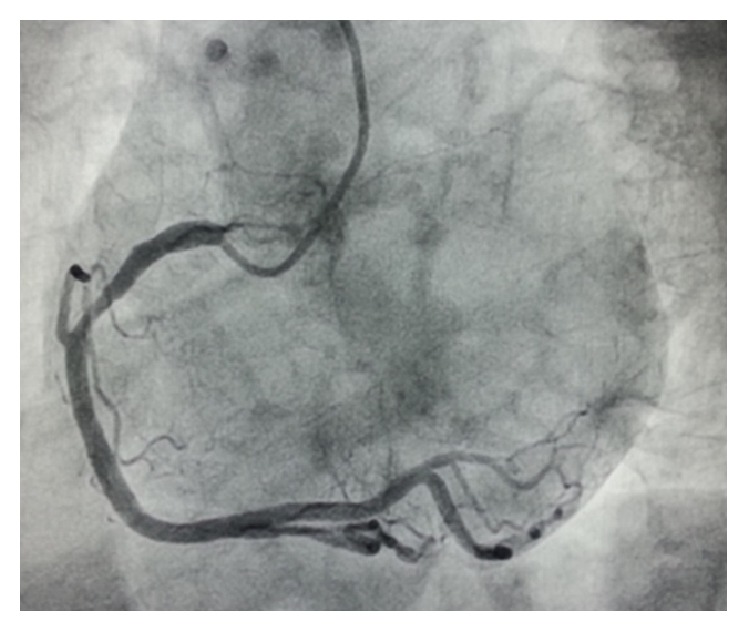
Super dominant RCA arising from right sinus in left anterior oblique view.

**Figure 5:**
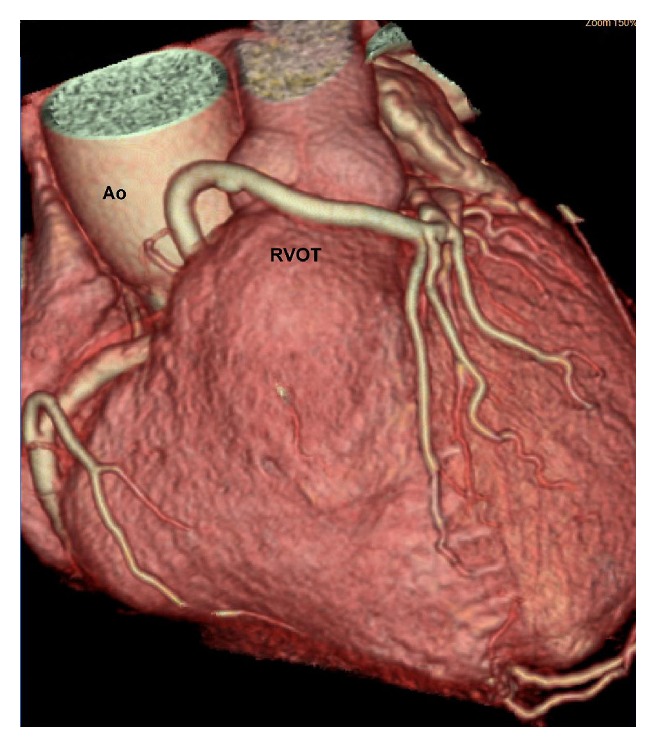
VR image showing ectopic LMCA with a prepulmonic course over the right ventricular outflow tract giving rise to LAD (Ao: aorta; RVOT: right ventricular outflow tract).

**Figure 6:**
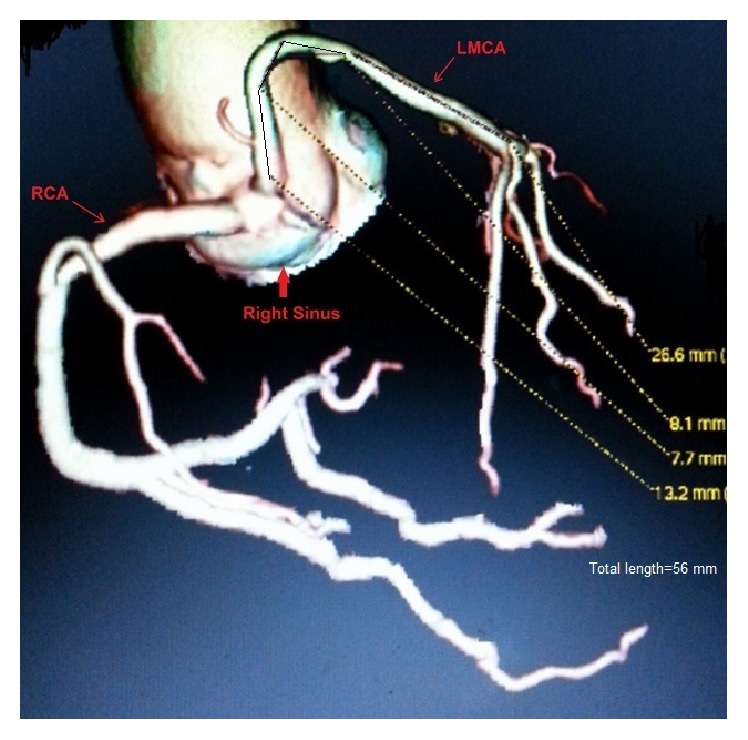
Superdominant RCA arising from right sinus in left anterior oblique view on MDCT.

Right coronary artery was visualized in its normal site which was super dominant (Figure 4). Both LMCA and RCA arising from right sinus could not be well delineated on single injection and therefore MDCT angiogram was done few days later revealing anomalous origin of LMCA from right sinus. On volume rendered (VR) image, it was going up after its origin and, with a reverse U-turn, it was coursing* anteriorly* over the right ventricular outflow tract (RVOT) finally giving rise to LAD and LCX (Figure 5). It was measuring 56 mm, thus confirming our diagnosis (Figure 6). LAD had a large septal branch and was terminating before reaching apex; thus it was type II.

## 3. Discussion

LMCA normally arises from left sinus of valsalva and has a diameter varying from 3 to 6 mm and length of 10 to 15 mm. The “short” and “long” main trunk (MT) were considered as variants in the length of the left coronary artery [3]. A short MT is considered to be one with a length of <5 mm [4]. Long LMCA is considered when it is over 15 mm in length though exact definition is lacking [5]. In their anatomical study by Reig and Petit, the longest left main trunk was reported to be 23 mm and the average length was 10.8 ± 5.5 mm [5]. LMCA originating from the right sinus of valsalva (RSV) is extremely rare, and it is incidentally found in approximately 0.017% of all coronary artery angiographies [6]. Left coronary artery originating from the right sinus of valsalva may have 4 courses: between the aortic root and the pulmonary artery (interarterial course), transseptal course (subpulmonic course), anterior course originating from the right ventricle (anterior or prepulmonic course), and posterior course regarding the aortic root (retroaortic course). Ectopic LMCA abnormality being quite uncommon, only few cases with long LMCA have been reported where maximum reported length is* 61 mm* but all had their usual origin from* left sinus *only [7–9]. A long LMCA is a coincidental finding. Though a short LMCA has been considered a risk factor in the development of coronary arteriosclerosis, no such relationship has been described between long LMCA and coronary arteriosclerosis [5]. It has been reported that short LMCA is associated with left dominance, whereas relatively long LMCA favours right coronary artery dominance which matches our case [10]. In our case, length of LMCA was almost* 61 mm* on conventional angiogram in different view and* 56 mm* on MDCT based on the calculation of the LMCA dimension by three intervention cardiologists and two radiologists and the average length was reported in order to nullify any magnification or calibration errors in measuring its dimensions by QCA software which also makes it unique as previous ones were based on conventional angiogram only. The discrepancy in length may be because QCA has a tendency of little overestimation. Another reason may be that tip of diagnostic catheter is difficult to be differentiated from the origin of left main artery, which might have been included in the measurement. We believe MDCT analysis to be more accurate than QCA. Whenever contemplating any intervention further, a guiding catheter with very good backup support should be chosen such as Amplatz Left or Ikari because hardware has to travel long distance before reaching its target. Another option may be the use of a buddy wire to venture more support of the guide catheter and facilitate the delivery of balloon and stent. In conclusion, to the best of our knowledge, our case has the longest angiographically documented* anomalous left main coronary artery* arising from right sinus of valsalva in the literature until now.
